# Identification of Single Nucleotide Polymorphisms Regulating Peripheral Blood mRNA Expression with Genome-Wide Significance: An eQTL Study in the Japanese Population

**DOI:** 10.1371/journal.pone.0054967

**Published:** 2013-01-24

**Authors:** Daimei Sasayama, Hiroaki Hori, Seiji Nakamura, Ryo Miyata, Toshiya Teraishi, Kotaro Hattori, Miho Ota, Noriko Yamamoto, Teruhiko Higuchi, Naoji Amano, Hiroshi Kunugi

**Affiliations:** 1 Department of Mental Disorder Research, National Institute of Neuroscience, National Center of Neurology and Psychiatry, Ogawahigashi, Kodaira, Tokyo, Japan; 2 Department of Psychiatry, Shinshu University School of Medicine, Matsumoto, Nagano, Japan; 3 DNA Chip Research Inc., Yokohama, Kanagawa, Japan; 4 National Center of Neurology and Psychiatry, Kodaira, Tokyo, Japan; 5 Core Research of Evolutional Science and Technology, Japan Science and Technology Agency, Chiyoda-ku, Tokyo, Japan; Massachusetts General Hospital, United States of America

## Abstract

Several recent studies have reported that expression quantitative trait loci (eQTLs) may affect gene expression in a cell-dependent manner. In the current study, a genome-wide eQTL analysis was performed in whole blood samples collected from 76 Japanese subjects. RNA microarray analysis was performed for 3 independent sample groups that were genotyped in a genome-wide scan. The correlations between the genotypes of 534,404 autosomal single nucleotide polymorphisms (SNPs) and the expression levels of 30,465 probes were examined for each sample group. The SNP-probe pairs with combined correlation coefficients of all 3 sample groups corresponding to *P*<3.1×10^−12^ (i.e., Bonferroni-corrected *P*<0.05) were considered significant. SNP-probe pairs with a high likelihood of cross-hybridization and SNP-in-probe effects were excluded to avoid false positive results. We identified 102 *cis*-acting and 5 *trans*-acting eQTL regions. The *cis*-eQTL regions were widely distributed both upstream and downstream of the gene, as well as within the gene. The eQTL SNPs identified were examined for their influence on the expression levels in lymphoblastoid cell lines by using a public database. The results showed that genetic variants affecting expression levels in whole blood may have different effects on gene expression in lymphoblastoid cell lines. Further studies are required to clarify how SNPs function in affecting the expression levels in whole blood as well as in other tissues.

## Introduction

Advances in high-throughput genotyping and gene expression platforms have enabled genome-wide analysis of gene expression quantitative trait loci (eQTLs), allowing investigation of both *cis* and *trans* effects. Previous eQTL studies have examined the association between genetic variants and gene expression levels in various biological samples, including human whole blood [1], [2], lymphocytes [3], the liver [4], and, primarily, in lymphoblastoid cell lines [5], [6]. Recently developed web tools such as SNPexp [7] and Genevar [8] have enabled analysis of the correlation between SNP genotypes in HapMap genotype data and genome-wide expression levels in lymphoblastoid cell lines. Development of such tools in other cell types is also anticipated, as a substantial fraction of eQTLs are cell type-specific [9], [10], [11], [12].

Despite these advances, several challenges still remain in the field of genome-wide eQTL research. The large number of gene expression traits and genomic loci requires enormous calculations, raising issues of computer efficiency and statistical power. Another challenge is the varying genetic backgrounds in study populations, which may be one of the causes of the poor reproducibility observed across studies. Furthermore, confounding variables, such as the time of day at which sampling was performed, may also affect gene expression patterns in peripheral blood [13]. In addition, microarray probes may contain one or more SNPs in the target sequence. These probes may cause hybridization differences due to sequence polymorphisms present in the mRNA region, resulting in the occurrence of false positive results [14]. Other probes may undergo cross-hybridization, also resulting in false positive results for *trans*-eQTLs. The large number of probes and SNPs cause difficulties in accounting for these confounding and influencing variables. A limited number of studies have overcome these methodological issues; therefore, further accumulation of data is required. Specifically, genome-wide eQTL data for Asian population is scarce [15].

Gene expression in whole blood could function as biomarkers for several disease conditions such as diabetes [16] and attention deficit hyperactivity disorder [17]. Elucidation of the genetic basis affecting such gene expression may be important in uncovering the etiological factors and pathophysiology of the diseases. Taking the aforementioned issues into consideration, we have examined the correlations between the genotypes of every SNP from a genome-wide scan and the expression levels of genes in the whole blood of Japanese individuals. To avoid the influence of batch effects, which is often ignored in eQTL studies, microarray data collected in different batches were first analyzed separately and then integrated. After strict corrections for multiple testing and exclusion of potential false-positive eQTLs, we investigated whether the SNPs found to have an effect on the expression levels in whole blood also influenced the expression levels in lymphoblastoid cell lines. Public data from the HapMap project of SNP genotypes and gene expression levels in lymphoblastoid cell lines were used for the analysis.

## Materials and Methods

Genomic DNA was collected from 24 subjects (13 men and 11 women, mean age [SD] = 39.9 [7.6] years) in sample group 1, 24 subjects in sample group 2 (12 men and 12 women, 34.1 [11.5] years), and 28 subjects (14 men and 14 women, 41.4 [11.8] years) in sample group 3. The blood samples of each of the 3 sample groups were collected at different times and the microarray data of each sample group were obtained separately. Approximately half of the subjects suffered from depressive disorder (11, 12, and 16 subjects in sample groups 1, 2, and 3, respectively), but all were physically healthy and without clinically significant systemic disease (e.g., malignant disease, diabetes mellitus, hypertension, renal failure, or endocrine disorders). Subjects were recruited from the outpatient clinic of the National Center of Neurology and Psychiatry Hospital, Tokyo, Japan, through advertisements in free local information magazines or through our website announcement. All the subjects were biologically unrelated Japanese individuals who resided in the same geographical area (western Tokyo). The study protocol was approved by the ethics committee at the National Center of Neurology and Psychiatry, Japan. Written informed consent was obtained from every subject after the study was explained to them.

Venous blood was collected between 1100 and 1200 h in PAXgene tubes (Qiagen, Valencia) from each subject and was incubated at room temperature for 24 h for RNA stabilization. RNA was extracted from whole blood according to the manufacturer’s guidelines by using the PAXgene Blood RNA System Kit (PreAnalytix GmbH, Hombrechtikon, Switzerland). The RNA was quantified by optical density readings at A260 nm by using the NanoDrop ND-1000 (Thermo Scientific, Rockford). Gene expression analysis was performed using Agilent Human Genome 4×44 K arrays (Agilent Technologies, Santa Clara). Raw signal data for each of the 3 independent sample groups were analyzed separately by the GeneSpring GX software (Agilent Technologies). Data were filtered according to the expression level for quality control to eliminate genes that were below the 20th percentile threshold. The expression value of each gene was normalized to the median expression value of all genes in each chip. A total of 30,465 probes were included in the analysis.

Genomic DNA was obtained from venous blood samples. Genotyping was performed by Riken Genesis (Yokohama, Japan) using the Illumina HumanOmni1-Quad BeadChip (Illumina, Inc., San Diego). A total of 713,495 autosomal SNPs were assessed for quality using the PLINK v1.07 software [18]. All SNPs with a call rate below 95%, a deviation from Hardy-Weinberg equilibrium at an error level of *P*<0.001, or a minor allele frequency of less than 10% were excluded. The remaining 534,404 SNPs were used for further analysis. RNA expression and DNA genotype data are available at NCBI’s Gene Expression Omnibus under accession number GSE42488.

Since RNA expression arrays of the 3 sample groups were performed at different times, the correlation between the genotype and expression levels was calculated separately in each sample group to avoid the influences of batch effects. The Pearson’s correlation coefficient (*r*) between the genotype (coded as 0, 1, or 2) and gene expression level was calculated for each of the 1.63×10^10^ SNP-expression probe pairs in the 3 sample groups. The correlation coefficients of the 3 sample groups were averaged according to the following equation [19]:


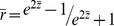


where 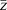 = Σ_i_ [1/2×ln{(1+*r*_i_)/(1−*r*_i_)}× n_i_]/Σ_i_ n_i_

n_i_ = the number of individuals in sample group i.

*r*_i_ = the correlation coefficient between the genotype and expression level in sample group i.

To minimize the possibility of false positives, the SNP-expression probe pairs with 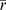 corresponding to a Bonferroni-corrected *P* value of <0.05 (i.e., uncorrected *P*<0.05/[30,465×534,404] = 3.1×10^−12^) were also examined using Spearman’s rank correlation in a similar method as described above. The SNP-probe pairs with Bonferroni-corrected *P* value of the average Spearman’s rank correlation <0.05 (i.e., uncorrected *P*<3.1×10^−12^) were considered significant.

To determine the potential for cross-hybridization of the probes, a BLAST search (http://blast.ncbi.nlm.nih.gov/Blast.cgi) was performed against the human genome by using the online Ensembl database. Probes with greater than 50% homology with other genomic regions were excluded.

Sequence polymorphisms in the mRNA region targeted by the microarray expression probes may cause hybridization differences due to SNP-in-probe effects. Therefore, SNP-probe pairs were excluded from the analysis if the 60-mer probe was mapped to a genomic location that contained a known SNP showing linkage disequilibrium (LD; r^2^>0.1) with the SNP of the SNP-probe pair.

We also examined whether the eQTL SNPs affecting the expression levels in whole blood also influence expression levels in lymphoblastoid cell lines. The SNPexp [7] software was used to retrieve public data from the HapMap project (release 23) of SNP genotypes and the gene expression levels in lymphoblastoid cell lines of 45 Japanese subjects. Pearson’s correlation coefficients were used to assess the influence of SNPs on expression levels in lymphoblastoid cell lines.

## Results

### Identification of eQTLs

**Figure 1:**
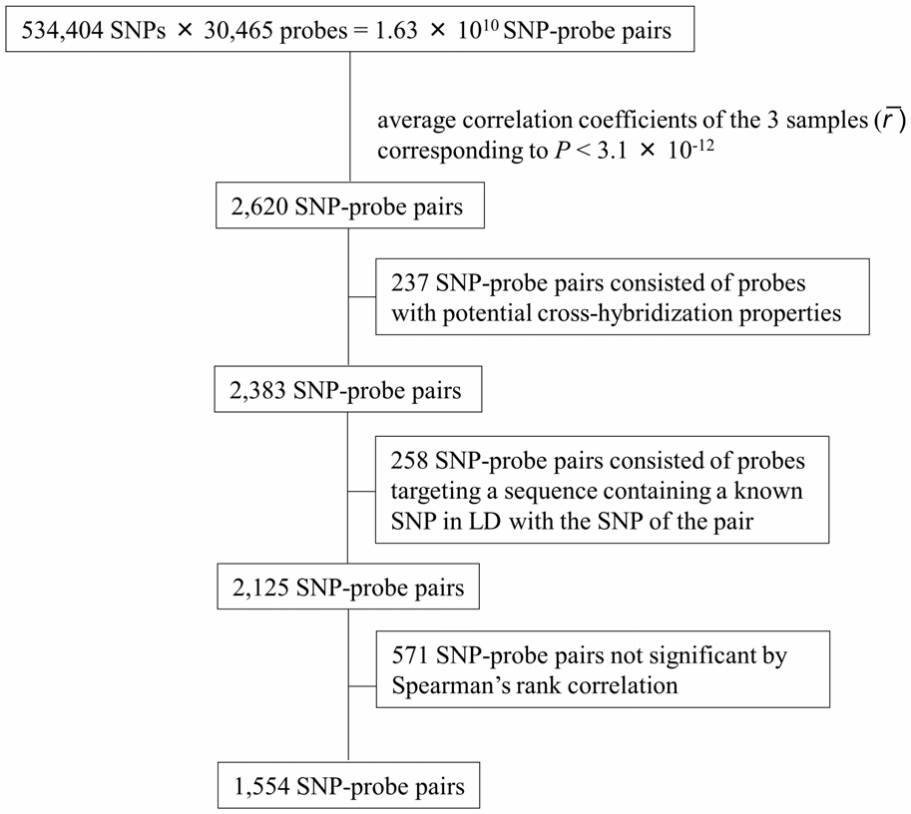
Procedure for selecting significant SNP-probe pairs. The procedure for selecting significant SNP-probe pairs is shown. SNP-probe pairs with a high likelihood of cross-hybridization and SNP-in-probe effects were excluded to exclude false positive results. The SNPs of the remaining 1,554 SNP-probe pairs were considered as eQTL SNPs.

**Figure 2:**
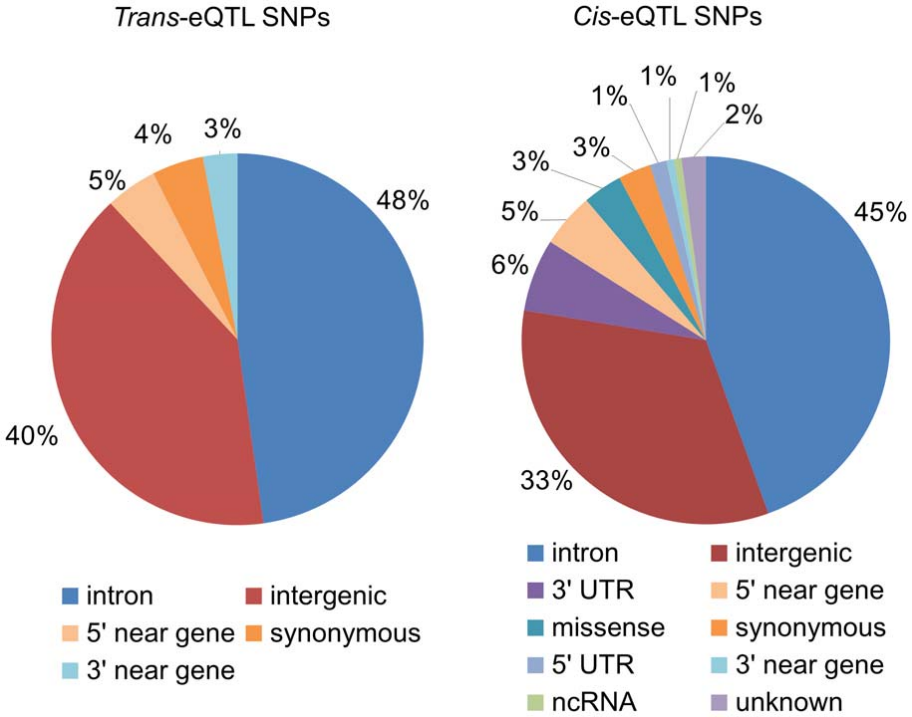
Functional types of the eQTL SNPs. The percentage of SNP types is shown for *cis-* and *trans-* eQTL SNPs.

The procedure used for SNP-probe pair selection (Figure 1) generated 1,554 pairs, which are listed in Table S1. These SNP-probe pairs consisted of 1,153 SNPs, defined as eQTL SNPs, and 185 probes. For 122 of these 185 probes, we could identify the corresponding gene from the HapMap database (Release 28). Since several of the probes targeted the same gene, the total number of genes identified was 107. As shown in Figure 2, the majority of the eQTL SNPs were located in intronic (45% and 48% for *cis*- and *trans*-eQTL SNPs, respectively) or intergenic (33% and 40% for *cis*- and *trans*-eQTL SNPs, respectively) regions.

Table S2 shows the names and properties of the 107 genes whose expression levels in whole blood were affected by SNPs. The SNPs affecting expression levels of the same gene were primarily in high LD with each other. Furthermore, investigation of combined Chinese and Japanese (CHB+JPT) panels from the 1000 Genomes Pilot 1 SNP data set and the HapMap release 22 data set showed a greater number of SNPs in high LD (r^2^>0.8) with the eQTL SNPs identified in the current study. Since the high intermarker correlations cause difficulties in determining which SNP is responsible for the regulation of gene expression, we defined the eQTL region of a gene as the genomic range in which the SNPs in LD (r^2^>0.8) with the eQTL SNPs of the gene are located. LD was determined by SNAP [20] using the population panel CHB+JPT from the 1000 Genomes Pilot 1 SNP data set and the HapMap release 22 data set.

### Locational Relationships between the eQTL and the Gene

Regarding the locational relationships between the eQTL and the gene, 102 of the eQTLs were *cis*-acting (within 1 Mb upstream or downstream of the gene), and 5 were *trans*-acting, of which 4 were located on a different chromosome from the gene that they influenced. When the genome was divided into 3 segments (i.e., upstream, intragenic, and downstream), 69 *cis*-acting eQTL regions covered multiple segments that included the intragenic segments, 13 were confined to upstream segments, 7 were confined to downstream segments, and 13 were confined to intragenic segments.

### Comparison of Results with Previously Reported Whole Blood eQTLs

We compared our results with those of the study by Fehrmann et al. [21], which performed a genome-wide eQTL analysis on 289,044 SNPs in whole blood expression data of 1,469 unrelated individuals from the United Kingdom and the Netherlands. The genotyping platform which they used (Illumina HumanHap300 platform) included only 24% of the 534,404 SNPs analyzed in the current study and 15% of the 1,153 eQTL SNPs identified in the current study. Therefore, 85% of the eQTL SNPs identified in the current study had not been identified by Fehrmann et al. [21], because they were not included in the Illumina HumanHap300 platform. On the other hand, 84% of the eQTL SNPs identified in the current study which were included in the Illumina HumanHap300 platform had also been identified as eQTL SNPs in their study. The high replication rate supports the robustness of our findings.

### Influence on Expression Levels in Lymphoblastoid Cell Lines

Next, we examined whether the eQTL SNPs affecting the expression levels in whole blood also influence expression levels in lymphoblastoid cell lines. We selected representative SNPs in eQTL regions and examined their effects on the expression of the corresponding gene in lymphoblastoid cell lines. The SNPs that showed the strongest correlation with the expression levels in whole blood for each eQTL region were selected for examination of the possible effects on expression levels in lymphoblastoid cell lines. If there were any additional eQTL SNPs in the same region that were not in LD with the selected SNP (r^2^<0.1), then one of the SNPs with the strongest correlation with the expression levels in whole blood was also selected. In the eQTL regions for *MICA*, *MICB*, *HLA-DRB5*, *HLA-DQB1*, and *HLA-DQA2*, 2 representative SNPs, which were not in significant LD with each other (r^2^<0.1), were selected. For other genes, the eQTL SNPs in the same eQTL region were in LD with each other (r^2^>0.1); therefore, 1 representative SNP was selected for each region. If the genotype data of the selected SNP were not available in the HapMap data, the SNP within the same eQTL region having the next strongest correlation with the expression levels in whole blood was selected.

Genotype and expression levels in lymphoblastoid cell lines were retrieved from public data for 45 Japanese individuals for 88 (86 *cis* and 2 *trans*) of the 112 representative SNPs. The average number of individuals with applicable data for genotype and the expression levels of lymphoblastoid cell lines in the 88 retrieved SNP-gene pairs was 43.8. The Pearson’s correlation coefficients between the eQTL SNPs and the expression levels of the corresponding genes in lymphoblastoid cell lines were calculated and have been shown in Table S3. A positive correlation coefficient indicates that the SNP has a similar effect on expression levels in whole blood and lymphoblastoid cell lines. Of the 86 *cis*-eQTL SNPs, 34 showed a significantly positive correlation, whereas 13 showed a significantly negative correlation with the expression levels of lymphoblastoid cell lines (FDR-corrected, *P*<0.05). None of the *trans*-eQTL SNPs identified in the current study significantly affected expression levels in lymphoblastoid cell lines.

### Functional Properties of the eQTL SNPs

We examined whether the regulatory effects of eQTL SNPs were caused by mutations in transcription factor-binding sites (TFBSs), splicing-affecting sites, or microRNA (miRNA)-binding sites. The proportion of SNPs in LD (r^2^>0.8) with a SNP predicted to be located on such sites was compared between the 37 eQTL SNPs affecting expression levels in both whole blood and lymphoblastoid cell lines; 49 eQTL SNPs affecting only whole blood expression levels; and 5,681 non-eQTL SNPs located within 100 kB of the 107 genes that were regulated by the eQTL SNPs identified in the current study. A web-based tool (FuncPred; http://snpinfo.niehs.nih.gov/snpinfo/snpfunc.htm) was used to predict the functional properties of the SNPs. As shown in Table 1, eQTL SNPs were more likely to be in LD with SNPs located on TFBSs, splicing-affecting sites, and miRNA-binding sites.

**Table 1:** Percentage of SNPs that are in linkage disequilibrium (r^2^>0.8) with a SNP predicted to be located on TFBS, splicing-affecting site, or miRNA binding site.

	TFBS	Splicing	miRNA binding site
eQTL SNPs affecting expression levels in both whole blood and LCLs (37 SNPs)	73.7% ‡	42.1% ‡	44.7% ‡
eQTL SNPs affecting expression levels in only whole blood (49 SNPs)	58.8% ‡	43.1%‡	29.4% †
non-eQTL SNPs (5,681 SNPs)	34.8%	17.3%	14.1%

The following abbreviations are used: TFBS, transcription factor binding site; miRNA, micro RNA; LCL, lymphoblastoid cell line.

† P<0.01,

‡ P<0.001: Significantly higher compared to non-eQTL SNPs (χ^2^ test).

### Cis-only Analysis

The small-effect eQTL SNPs are likely to have remained undetected in the present study due to the strict correction procedures for multiple testing. In order to reduce the number of unreported *cis*-eQTL SNPs, we also performed *cis*-only analysis by examining only SNPs 1 Mb upstream or downstream of the targeted gene. A total of 955,370 SNP-probe pairs were examined, and those with an average Pearson’s correlation (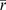) of the 3 sample groups corresponding to *P*<5.23×10^−9^ (i.e., Bonferroni-corrected *P*<0.05) were considered significant. As shown in Table S4, the *cis*-only analysis resulted in 3,883 SNP-probe pairs consisting of 3,161 SNPs and 347 probes.

### The Influence of Depressive Disorder on Gene Expression Regulation

In order to investigate whether depressive disorder was a major confounding factor for gene expression regulation, we calculated the Spearman’s correlation coefficients separately in depressed and non-depressed subjects. All the 1,554 SNP-probe pairs identified as eQTL in the present study achieved high correlations for both depressed and non-depressed subjects (average Spearman’s correlation of the 3 sample groups 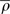
>0.4, FDR-corrected *P*<0.01 in non-depressed subjects and 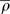
>0.5, FDR-corrected *P*<0.005 in depressed subjects for all 1,554 SNP-probe pairs).

## Discussion

To our knowledge, this is the first genome-wide eQTL study in Asian subjects that examined the association of SNPs with expression levels in whole blood. The genome-wide investigation uncovered 1,153 SNPs affecting gene expression levels in human whole blood. Although the number of eQTL regions identified in the current study was relatively small, the likelihood of false positives is low because of the strict correction procedures for multiple testing and exclusion of SNPs with potential cross-hybridization or SNP-in-probe effects.

Since SNPs in strong LD with a SNP directly responsible for regulating gene expression levels are also correlated with gene expression levels, it is difficult to determine which SNP is the causative one. We assumed that the genetic regulatory locus would be included in the eQTL region, defined as the genomic range in which the SNPs in LD (r^2^>0.8) with the eQTL SNPs identified in our study are found. Although the numerous SNPs in LD with each other hindered the identification of the responsible SNP, the locations of the eQTL regions indicated that eQTLs are widely distributed both upstream and downstream of the gene, as well as within the gene.

The current study showed that several of the SNPs affecting the expression levels of a gene in whole blood also influenced the expression levels of the same gene in lymphoblastoid cell lines. A recent study by Powell et al. [22] has shown that the genetic control mechanisms of gene expression in whole blood and lymphoblastoid cell lines are largely independent. Despite the evidence of low genetic correlation of regulatory variation averaged across the genome, our results suggest that a subset of eQTLs commonly affect expression levels in whole blood and lymphoblastoid cell lines. Conversely, our findings suggest that some of the whole blood eQTL SNPs do not regulate expression levels in lymphoblastoid cell lines. This is in line with a previous study that reported that 69–80% of the identified regulatory variants operated in a cell type-specific manner [9]. Compared to SNPs affecting only expression levels in whole blood, higher, although not statistically significant, proportion of SNPs affecting expression levels in both whole blood and lymphoblastoid cell lines were in LD with SNPs located on TFBSs and miRNA-binding sites. The finding suggests that these functional properties affect expression levels across multiple cell types.

Intriguingly, 13 of the 88 eQTL SNPs in whole blood were observed to have opposite effects on expression levels in whole blood and lymphoblastoid cell lines. Dimas et al. [9] compared gene expression variation in fibroblasts, lymphoblastoid cell lines, and T cells and reported that the same directional effect in each cell type was observed for eQTLs shared between multiple cell types. However, 2 recently published studies reported that some eQTL SNPs have opposite allelic effects on gene expression in the liver, adipose tissue, skeletal muscle [10], or in B cells and monocytes [11]. Our findings also suggest the possibility that some SNPs may exert opposite effects on gene expression in different cell types. However, an alternative explanation may be that the eQTL SNPs identified may function to alter the splicing of the mRNA. Since the gene expression microarray platform used in the previous eQTL study examining LCL expression levels in Japanese subjects was different from ours, the different probes may have detected different splicing variants, resulting in seemingly opposite allelic effects. A comparison using the same platform would be necessary to uncover cell-specific effects on expression levels.

The strength of the current study is that a relatively homogeneous Japanese population was used, which may have minimized the effects of differential genetic backgrounds. The major limitation of the current study is that the conservative corrections for multiple testing may have missed a large proportions of eQTL SNPs. Increasing power allows better detection of weaker and more distantly located *cis*-regulatory elements [23]. Greater than 82% of the significant eQTL-probe pairs identified in the current study had *P*<3.1×10^−13^, which far exceeded the predetermined significance level (*P*<3.1×10^−12^). Our findings should not be generalized to more weakly associated eQTLs since they may have different regulatory mechanisms. Another limitation is that approximately half of the samples were collected from patients with a depressive disorder. However, analyzing healthy and depressive subjects separately also resulted in achieving high correlations (FDR-corrected *P*<0.01) for all the 1,554 SNP-probe pairs identified in the current study. Therefore, it is unlikely that depressive disorder has a major impact on gene expression regulation of the identified eQTL SNPs. Further investigation on the influence of depressive symptoms on gene expression levels is underway using a larger sample size.

In summary, we have presented the results on genome-wide investigations of SNPs affecting the expression levels in whole blood. Both *cis*-acting and *trans*-acting eQTL SNPs were identified for a total of 107 genes. The eQTL regions were widely distributed upstream, downstream, and within the gene sequence. The findings of this study are valuable if gene expression levels in whole blood are used as biomarkers for disease conditions. Gene expression levels and their connection with disease-associated SNPs may lead to a better understanding of genetic predisposition to disease and may be used to predict disease susceptibility. Further studies are required to clarify how SNPs function in affecting the expression levels in whole blood as well as in other tissues.

## Supporting Information

Table S1 **Significant SNP-probe pairs.** The SNP-probe pair selection procedure generated 1,554 significant pairs, consisted of 1,153 SNPs, defined as eQTL SNPs, and 185 probes.
(XLSX)

Table S2 **Genes whose expression levels in whole blood are affected by SNPs.** The names and properties of the 107 genes whose expression levels in whole blood were affected by SNPs are shown.
(XLSX)

Table S3 **The Pearson’s correlation coefficients between the eQTL SNPs and the expression levels of the corresponding genes in lymphoblastoid cell lines.** A positive correlation coefficient indicates that the SNP has a similar effect on expression levels in whole blood and lymphoblastoid cell lines. Of the 86 *cis*-eQTL SNPs, 34 showed a significantly positive correlation, whereas 13 showed a significantly negative correlation with the expression levels of lymphoblastoid cell lines (FDR-corrected, *P*<0.05).
(XLSX)

Table S4 **The results of the** ***cis*** **-only analysis.** The *cis*-only analysis resulted in 3,883 SNP-probe pairs consisting of 3,161 SNPs and 347 probes.
(XLSX)
